# Supplementary material for: Levels of additive genetic variation vary substantially between species
Source: PLoS Biol. 2026 Jun 18;24(6):e3003819. doi: 10.1371/journal.pbio.3003819 (PMC13278422; doi:10.1371/journal.pbio.3003819)
Supplement: S1 Appendix — (PDF) [file pbio.3003819.s002.pdf]

Appendix 1

In order to estimate the proportion of heritability and evolvability estimates that we have sampled we conducted an additional analysis using the Web of Science (WoS). An initial search for the keyword "Heritability" (1992-2022) returned 41,471 publications. Because heritability is used across many disciplines in ways that do not correspond to the quantitative genetic definition relevant to our study (i.e., the variance-standardised additive genetic variance), an initial category level filter ("Web of Science Categories") was applied, removing categories clearly unrelated to quantitative genetics in wild or non-domesticated populations, reducing the results to 12,461 publications.

We then removed journals ("Publication Titles") whose titles indicated an exclusive focus on humans, domesticated species, or crops, resulting in 10,667 publications. This step was necessary as our aim is to characterise heritability and evolvability in populations experiencing natural or near-natural ecological conditions. Finally, restricting the search to original research articles ("Document Type") removed reviews and book chapters, which often compile estimates from primary studies that would already be captured by our search strategy resulting in 9,614 publications.

(Web of Science Query Link: https://www.webofscience.com/wos/woscc/summary/460ed184-725f-4964-8bbc-3cd38fe1bb2b-01ac40b162/relevance/1)

To estimate what proportion of these 9,614 filtered publications might contain relevant estimates, we applied two complementary approaches. First, for the four focal journals included in our main compilation (Journal of Evolutionary Biology, Evolution, Heredity, and Proceedings of the Royal Society B), we know the proportion of publications that yielded relevant estimates (Figure). These journals were chosen precisely because they publish a high concentration of relevant publications, so the mean inclusion rate for these journals (~58%) is likely to overestimate the proportion of relevant publications across the broader literature.

Second, to obtain a more conservative estimate, we screened all 740 PLOS ONE results returned by the same WoS search at the title level. This is the journal with the largest number of results for our search. Because PLOS ONE has a broad editorial scope it provides a more representative sample of the heterogeneity present in the filtered pool. Of the 740 results, 581 (75.5%) were excluded at the title stage, leaving 159 (21.5%) potentially relevant publications. This 21.5% is itself an upper bound, as screening was only done based on title and therefore did not assess whether relevant were reported.

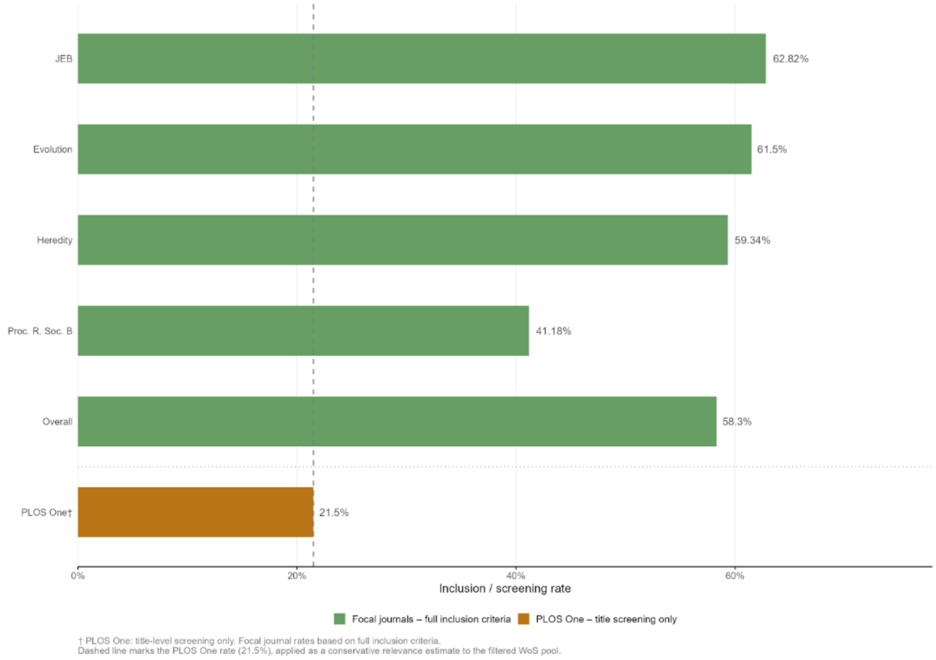

Figure 1: Inclusion rates across focal journals and the broader WoS pool. Bars show the proportion of screened articles meeting inclusion criteria for each of the four focal journals (Journal of Evolutionary Biology, Evolution, Heredity, and Proceedings of the

Royal Society B) and overall (green). The PLOS One rate (orange) reflects title-level screening only (21.5%) and is shown as a conservative estimate of relevance in the filtered WoS pool; this rate is indicated by the dashed vertical line. Overall inclusion across focal journals was 58.3%.

Applying this conservative 21.5% relevance rate to the 8,592 publications outside of the four focal journals yields an estimated 1,847 potentially relevant publications. Adding the 669 confirmed inclusions from the focal journals gives an estimated total pool of 2,516 relevant publications. After filtering out humans, domesticated species and crops our dataset contains 469 publications, representing approximately 19% of the total relevant publications. This is the proportion of estimates that we have sampled if we assume that the number of estimates in the unsampled journals is similar to that in the sampled journals.

As 92% of evolvability estimates are accompanied with heritabilities, and assuming this pattern is representative of the broader literature, we can extend these findings to evolvability as a reasonable proxy for the proportion of estimates captured.

We note that this screening analysis focuses on the keyword “Heritability”. Although, our main compilation also searched for “Evolvability”, this term is even more heterogeneous in usage and far less frequently reported. In our original compilation, the keyword “Evolvability” returned on average only 46 publications per focal journal, of which ~4 per journal were relevant. For this reason, and because the majority of both heritability and evolvability estimates were captured using “Heritability” as a keyword, we based the screening analysis on heritability alone.

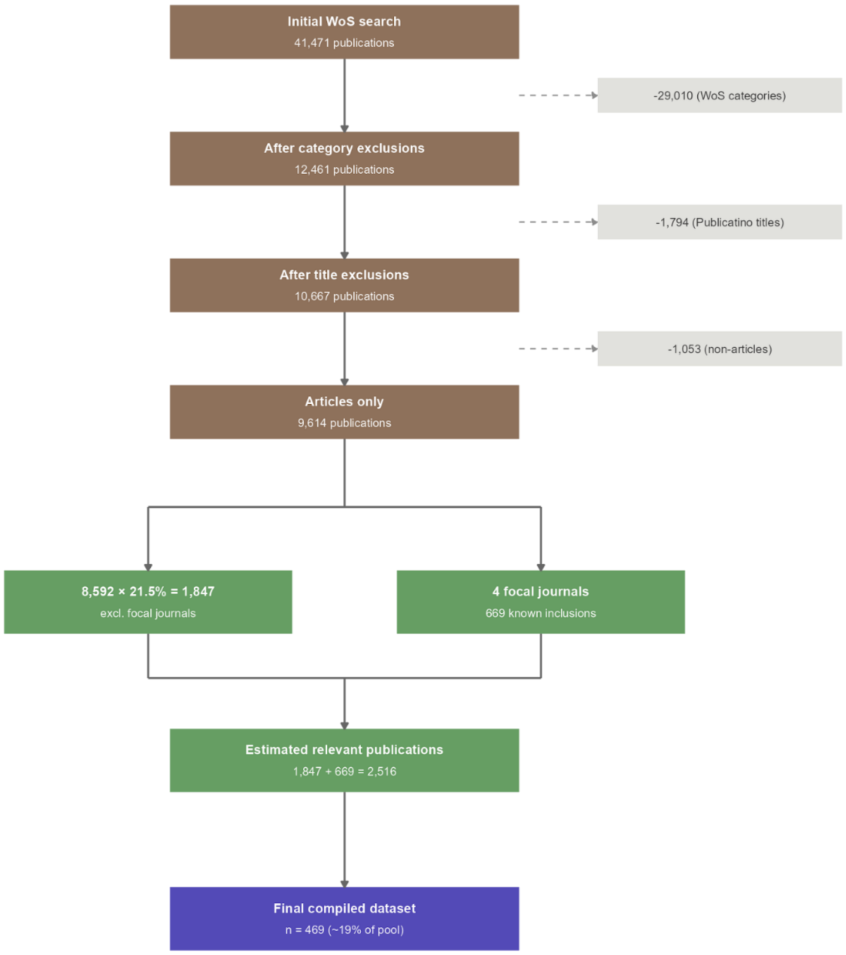

Figure 2: Flowchart of literature search and dataset compilation.
