# Supplementary material for: Levels of additive genetic variation vary substantially between species
Source: PLoS Biol. 2026 Jun 18;24(6):e3003819. doi: 10.1371/journal.pbio.3003819 (PMC13278422; doi:10.1371/journal.pbio.3003819)
Supplement: S2 Appendix — (PDF) [file pbio.3003819.s003.pdf]

Appendix 2

Table A: Data Summary. Number of evolvability and heritability estimates by fixed effect level

Fixed Effect	Level	n (I_A)	n(h^2)
Dimension	Linear	844	1059
	Quadratic	70	64
	Cubic	293	300
	Count	273	257
	Time	140	243
	Other	232	1051
Environment	Natural	484	755
	Lab	1119	1806
	Field/lab	249	413
Method	Animal model	1095	1582
	Mid-parent-offspring	62	162
	Half-sib	344	507
	Full-sib	101	236
	Single-parent-offspring	152	355
	Realized	1	1
	Clonal	97	131
Trait Type	Morphology	1258	1955
	Life-History	207	439
	Fitness	72	46
	Behaviour	82	147
	Physiological	233	387

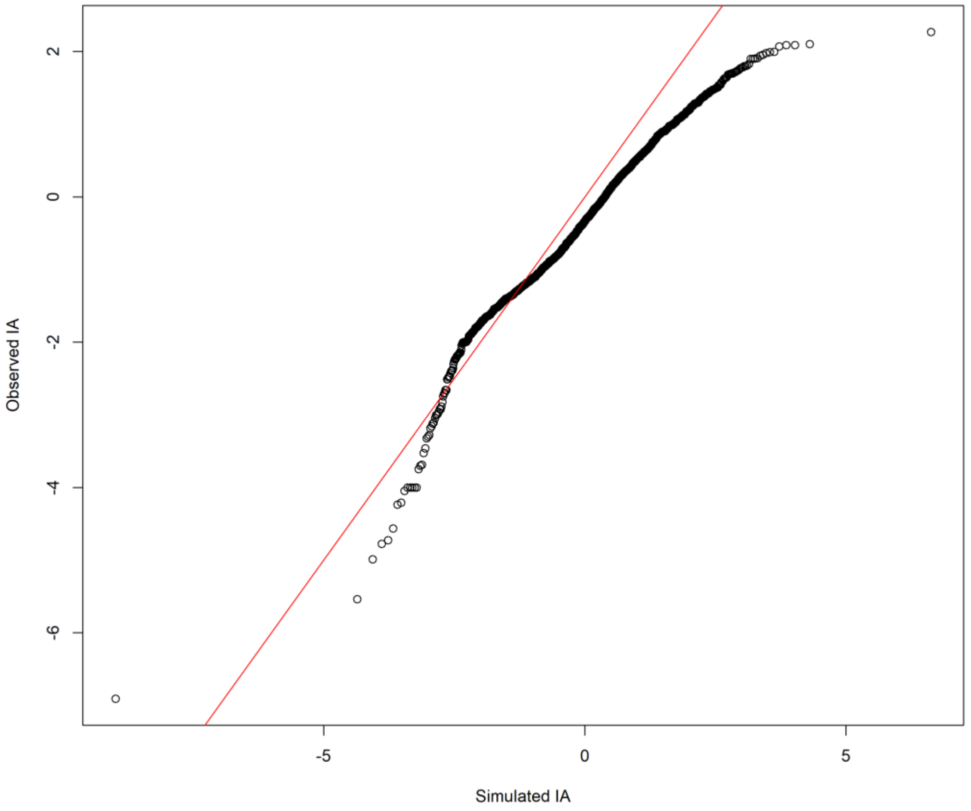

Fig. A: QQplot for visual inspection of model fit. Simulated I_A values using the simulate() function plotted in a qqplot against the observed I_A values. The data underlying this figure can be found in the data repository accompanying this paper under the DOI: https://doi.org/10.5281/zenodo.20021814

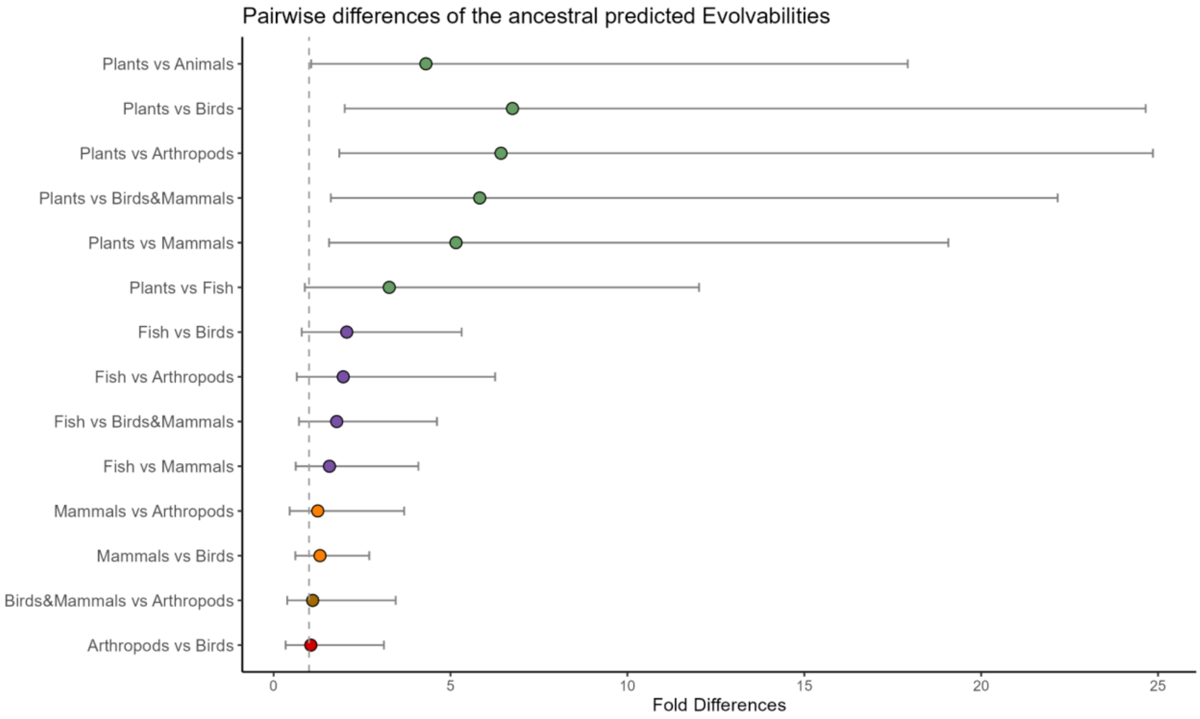

Fig. B: The difference in ancestral evolvability between groups of animals and plants. Each point represents the posterior mean difference between two ancestral nodes of the phylogeny, back-transformed to the original data scale. Horizontal bars indicate the 95% credible intervals. The dashed line at 1 indicates no difference between groups. Differences greater than 1 indicate that the first node (first-mentioned group in the y-axis) has a x-fold higher predicted evolvability than the second node. All comparisons are plotted so that the means are positive. The data underlying this figure can be found in the data repository accompanying this paper under the DOI: https://doi.org/10.5281/zenodo.20021814

Table B: Summary of the random and fixed effects for the interspecific variation model of evolvability with the inclusion of the taxonomic (Plant/Animal) fixed effect. Random effects as the proportion of variance explained by them and the p-values of an omnibus test for the fixed effects.

**Random Effect**	**mean**	**l-95%**	**u-95%**	**Percentage variance explained**
**Phylogeny**	0.590	0.105	1.143	40% [17, 63]
**Non-Phylogenetic**	0.012	2e-09	0.048	1.0% [0, 4.0]
**Publication_ID**	0.182	0.118	0.253	14% [7.5, 22]
**Trait_2**	0.176	0.121	0.233	13% [7.1, 19]
**Residual**	0.424	0.391	0.462	32% [21, 45]

<table>
<tr><th></th><th>mean</th><th>l-95%</th><th>u-95%</th><th>pMCMC</th><th>P(>x^2)</th></tr>
<tr><td>Intercept</td><td>-0.769</td><td>-1.899</td><td>0.534</td><td>0.216</td><td></td></tr>
<tr><td>Method: clonal</td><td>-0.092</td><td>-0.534</td><td>0.330</td><td>0.688</td><td rowspan="7">0.011</td></tr>
<tr><td>Method: full-sib</td><td>0.611</td><td>0.226</td><td>1.030</td><td>0.003</td></tr>
<tr><td>Method: half-sib</td><td>0.033</td><td>-0.203</td><td>0.286</td><td>0.810</td></tr>
<tr><td>Method: mid-parent-offspring</td><td>-0.401</td><td>-0.782</td><td>-0.034</td><td>0.040</td></tr>
<tr><td>Method: realized</td><td>-0.478</td><td>-2.653</td><td>1.417</td><td>0.643</td></tr>
<tr><td>Method: single-parent-offspring</td><td>0.088</td><td>-0.192</td><td>0.367</td><td>0.553</td></tr>
<tr><td>Trait type: behaviour</td><td>0.728</td><td>0.404</td><td>1.048</td><td><4e-04</td><td rowspan="4"><4e-04</td></tr>
<tr><td>Trait type: fitness</td><td>-0.071</td><td>-0.700</td><td>0.604</td><td>0.842</td></tr>
<tr><td>Trait type: life history</td><td>0.027</td><td>-0.238</td><td>0.305</td><td>0.838</td></tr>
<tr><td>Trait type: physiology</td><td>0.190</td><td>-0.031</td><td>0.453</td><td>0.123</td></tr>
<tr><td>Dimension: quadratic</td><td>0.220</td><td>-0.076</td><td>0.532</td><td>0.155</td><td rowspan="5"><4e-04</td></tr>
<tr><td>Dimension: cubic</td><td>0.544</td><td>0.356</td><td>0.764</td><td><4e-04</td></tr>
<tr><td>Dimension: time</td><td>0.233</td><td>-0.072</td><td>0.552</td><td>0.148</td></tr>
<tr><td>Dimension: count</td><td>0.531</td><td>0.297</td><td>0.779</td><td><4e-04</td></tr>
<tr><td>Dimension: other</td><td>0.270</td><td>0.024</td><td>0.520</td><td>0.031</td></tr>
<tr><td>n_fixed</td><td>0.010</td><td>-0.029</td><td>0.050</td><td>0.642</td><td>0.645</td></tr>
<tr><td>n_random</td><td>0.093</td><td>0.004</td><td>0.179</td><td>0.036</td><td>0.038</td></tr>
<tr><td>Environment: lab</td><td>0.167</td><td>-0.086</td><td>0.427</td><td>0.213</td><td rowspan="2">0.002</td></tr>
<tr><td>Environment: field/lab</td><td>-0.324</td><td>-0.671</td><td>0.002</td><td>0.062</td></tr>
<tr><td>Taxonomic Group: Plants</td><td>0.729</td><td>-1.456</td><td>2.520</td><td>0.417</td><td>0.453</td></tr>
</table>

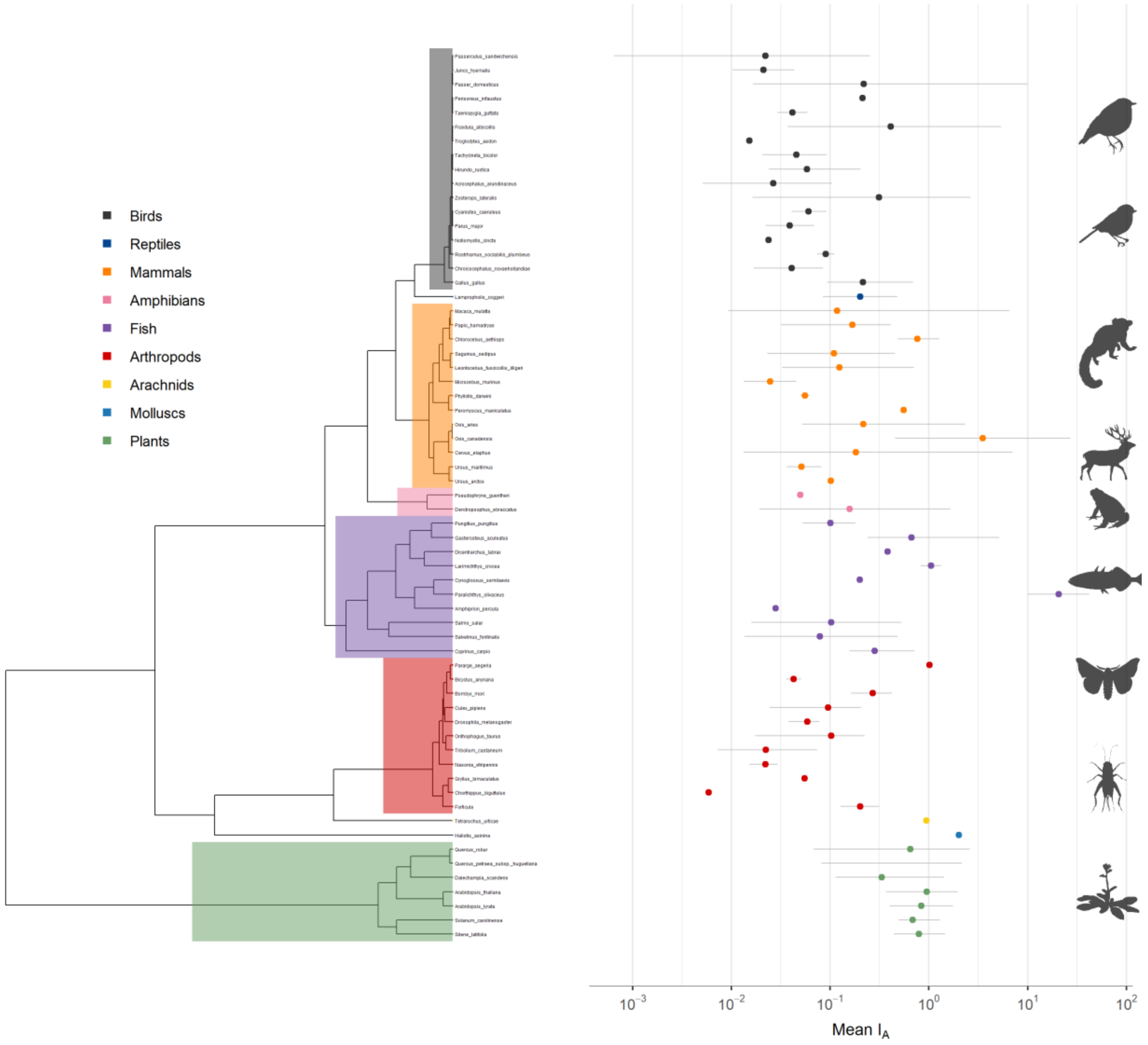

Fig. C: Mean I_A for each species for linear morphological traits which were estimated using an animal model. Organism silhouettes were obtained from PhyloPic (https://www.phylopic.org) and are in the public domain (CC0 1.0 Universal Public Domain Dedication or Public Domain Mark 1.0). The data underlying this figure can be found in the data repository accompanying this paper under the DOI: https://doi.org/10.5281/zenodo.20021814

Table C: Summary of the random and fixed effects for the interspecific variation model of evolvability. Random effects as the proportion of variance explained by them and the p-values of an omnibus test for the fixed effects.

**Random Effect**	**mean**	**l-95%**	**u-95%**	**Percentage variance explained**
**Phylogeny**	0.530	0.135	1.011	38% [15, 58]
**Non-Phylognetic**	0.012	8e-09	0.045	1.0% [0, 3.9]
**Publication_ID**	0.182	0.123	0.251	14% [7.8, 22]
**Trait_2**	0.177	0.123	0.233	14% [7.9, 20]
**Residual**	0.423	0.388	0.459	33% [23, 45]

	**mean**	**l-95%**	**u-95%**	**pMCMC**	**$P(>x^2)$**
**Intercept**	-0.443	-1.294	0.470	0.274	
**Method: clonal**	-0.100	-0.536	0.367	0.643	0.012
**Method: full-sib**	0.601	0.195	0.987	0.005	
**Method: half-sib**	0.037	-0.220	0.276	0.790	
**Method: mid-parent-offspring**	-0.404	-0.771	-0.034	0.035	
**Method: realized**	-0.503	-2.757	1.490	0.623	
**Method: single-parent-offspring**	0.092	-0.185	0.383	0.528	
**Trait type: behaviour**	0.717	0.388	1.025	<4e-04	<4e-04
**Trait type: fitness**	-0.055	-0.748	0.555	0.866	
**Trait type: life history**	0.028	-0.238	0.313	0.840	
**Trait type: physiology**	0.191	-0.071	0.415	0.140	
**Dimension: quadratic**	0.223	-0.082	0.544	0.173	<4e-04
**Dimension: cubic**	0.541	0.335	0.740	<4e-04	
**Dimension: time**	0.234	-0.108	0.542	0.143	
**Dimension: count**	0.531	0.277	0.772	<4e-04	
**Dimension: other**	0.267	0.009	0.519	0.043	
**n_fixed**	0.010	-0.029	0.048	0.600	0.601
**n_random**	0.092	0.007	0.177	0.033	0.040
**Environment: lab**	0.172	-0.078	0.414	0.162	0.003
**Environment: field/lab**	-0.305	-0.644	0.035	0.080	

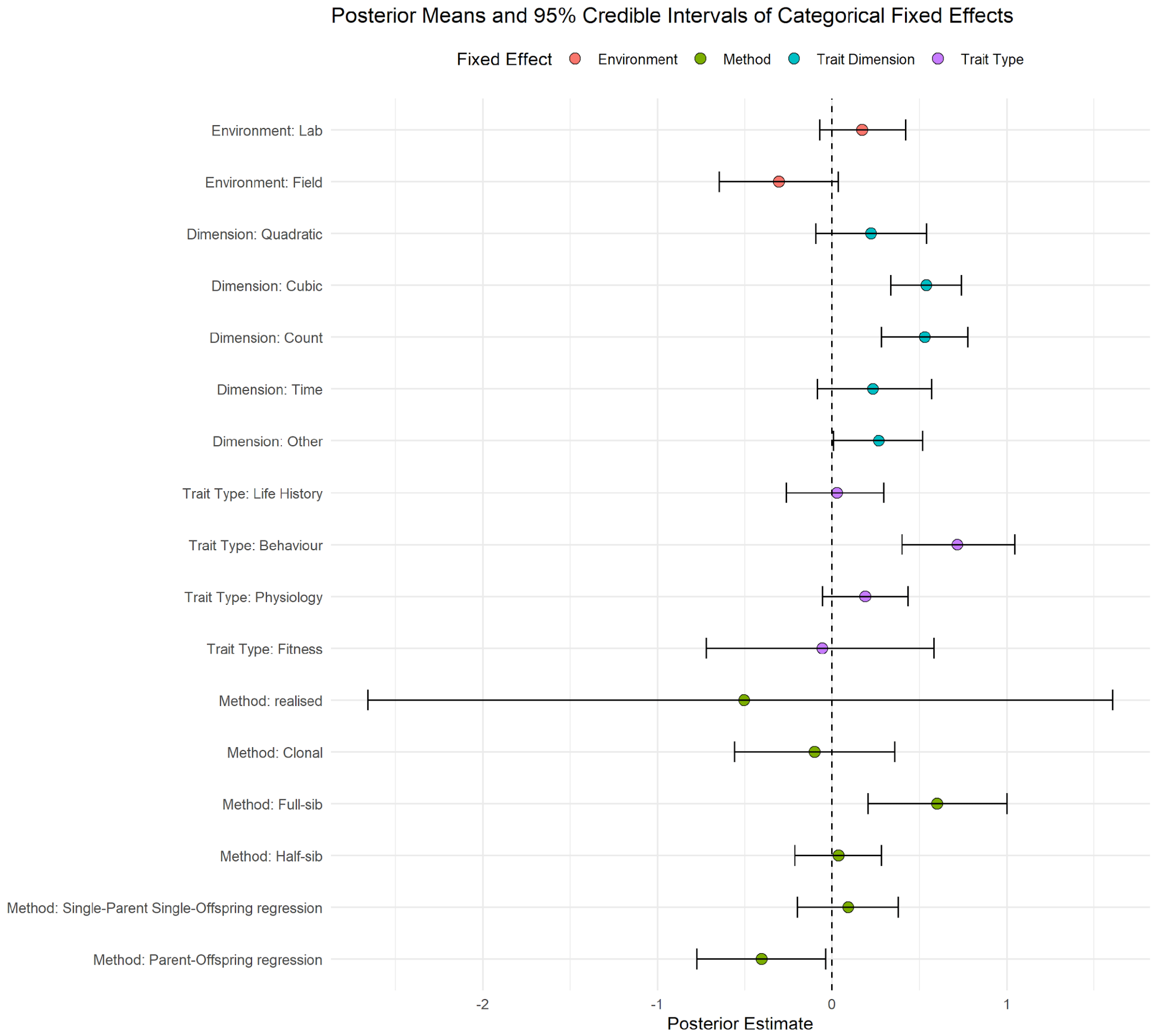

Fig. D: The posterior estimate for each fixed effect in the statistical model of I_A, along with its 95% credibility intervals; all estimates are deviations from the default model of a linear morphological trait estimated using an animal model from a population in the wild. The data underlying this figure can be found in the data repository accompanying this paper under the DOI: https://doi.org/10.5281/zenodo.20021814

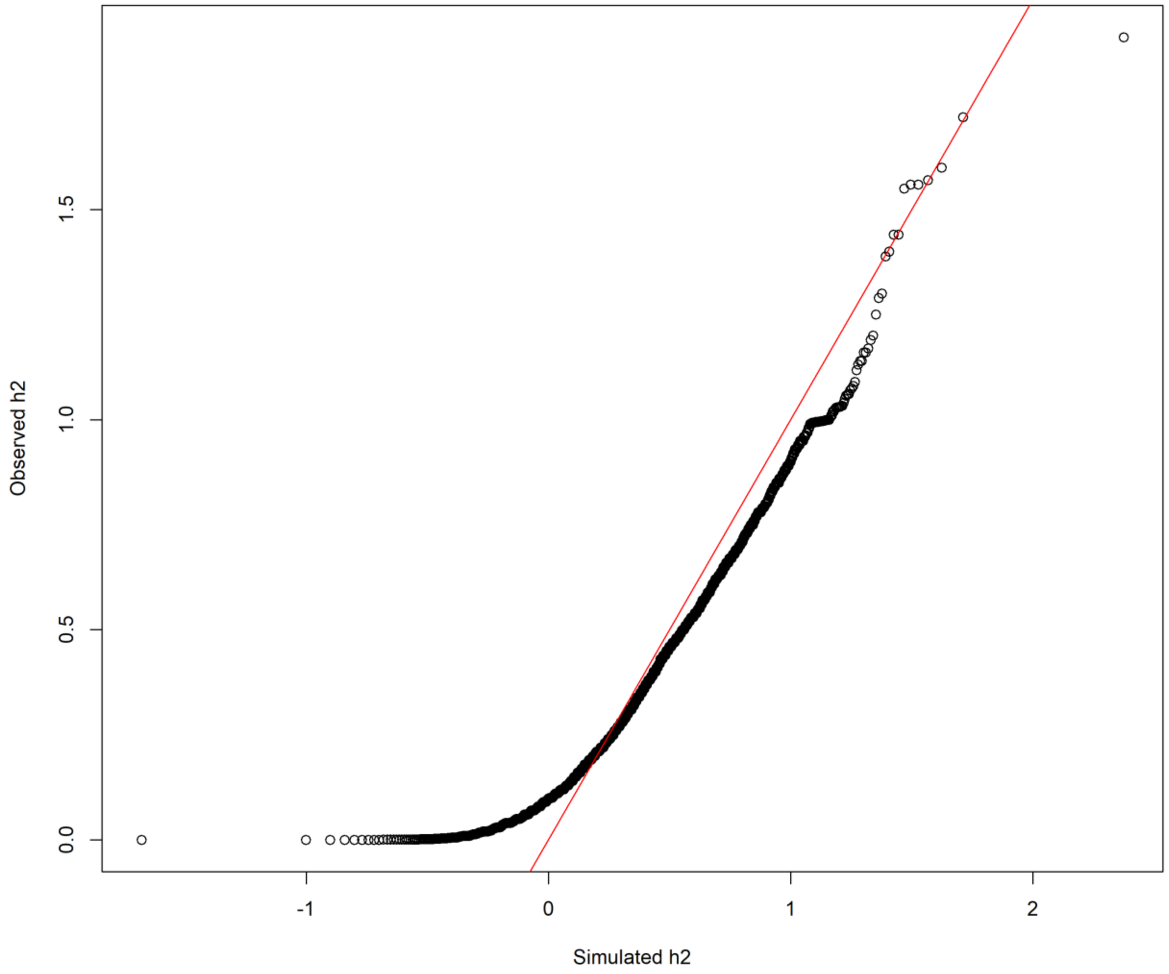

Fig. E: QQplot for visual inspection of model fit. Simulated h^2 values using the simulate() function plotted in a qqplot against the observed h^2 values. The data underlying this figure can be found in the data repository accompanying this paper under the DOI: https://doi.org/10.5281/zenodo.20021814

Table D: Summary of the random and fixed effects for the interspecific variation model of heritability. random effects as the proportion of variance explained by them and the p-values of an omnibus test for the fixed effects.

Random Effect	mean	l-95%	u-95%	Percentage variance explained	
**Phylogeny**	0.034	4e-09	0.090	28% [0.35, 57]	
**Non-Phylogenetic**	0.004	1e-08	0.010	4.4% [0, 12]	
**Publication_ID**	0.023	0.017	0.029	22% [11, 31]	
**Trait_2**	0.004	0.002	0.006	4.2% [1.7, 6.8]	
**Residual**	0.042	0.039	0.044	41% [25, 55]	
	**mean**	**l-95%**	**u-95%**	**pMCMC**	**P(>x^2)**
**Intercept**	0.326	0.110	0.557	0.013	
**Method: clonal**	-0.026	-0.136	0.092	0.668	<4e-04
**Method: full-sib**	0.207	0.108	0.300	<4e-04	
**Method: half-sib**	-0.004	-0.075	0.063	0.906	
**Method: mid-parent-offspring**	0.070	-0.013	0.149	0.094	
**Method: realized**	0.075	-0.492	0.621	0.799	
**Method: single-parent-offspring**	0.091	0.030	0.158	0.007	
**Trait type: behaviour**	-0.101	-0.159	-0.032	0.003	<4e-04
**Trait type: fitness**	-0.147	-0.284	-0.013	0.033	
**Trait type: life history**	-0.038	-0.081	0.007	0.087	
**Trait type: physiology**	-0.075	-0.117	-0.032	0.001	
**Dimension: quadratic**	-0.015	-0.088	0.054	0.684	0.012
**Dimension: cubic**	-0.015	-0.056	0.030	0.495	
**Dimension: time**	-0.017	-0.071	0.040	0.533	
**Dimension: count**	-0.053	-0.100	-0.001	0.042	
**Dimension: other**	-0.060	-0.094	-0.027	0.001	
**n_fixed**	-0.008	-0.018	0.002	0.118	0.115
**n_random**	-0.023	-0.043	-0.003	0.026	0.025
**Environment: lab**	0.111	0.042	0.179	0.003	0.002
**Environment: field/lab**	0.036	-0.050	0.118	0.401	

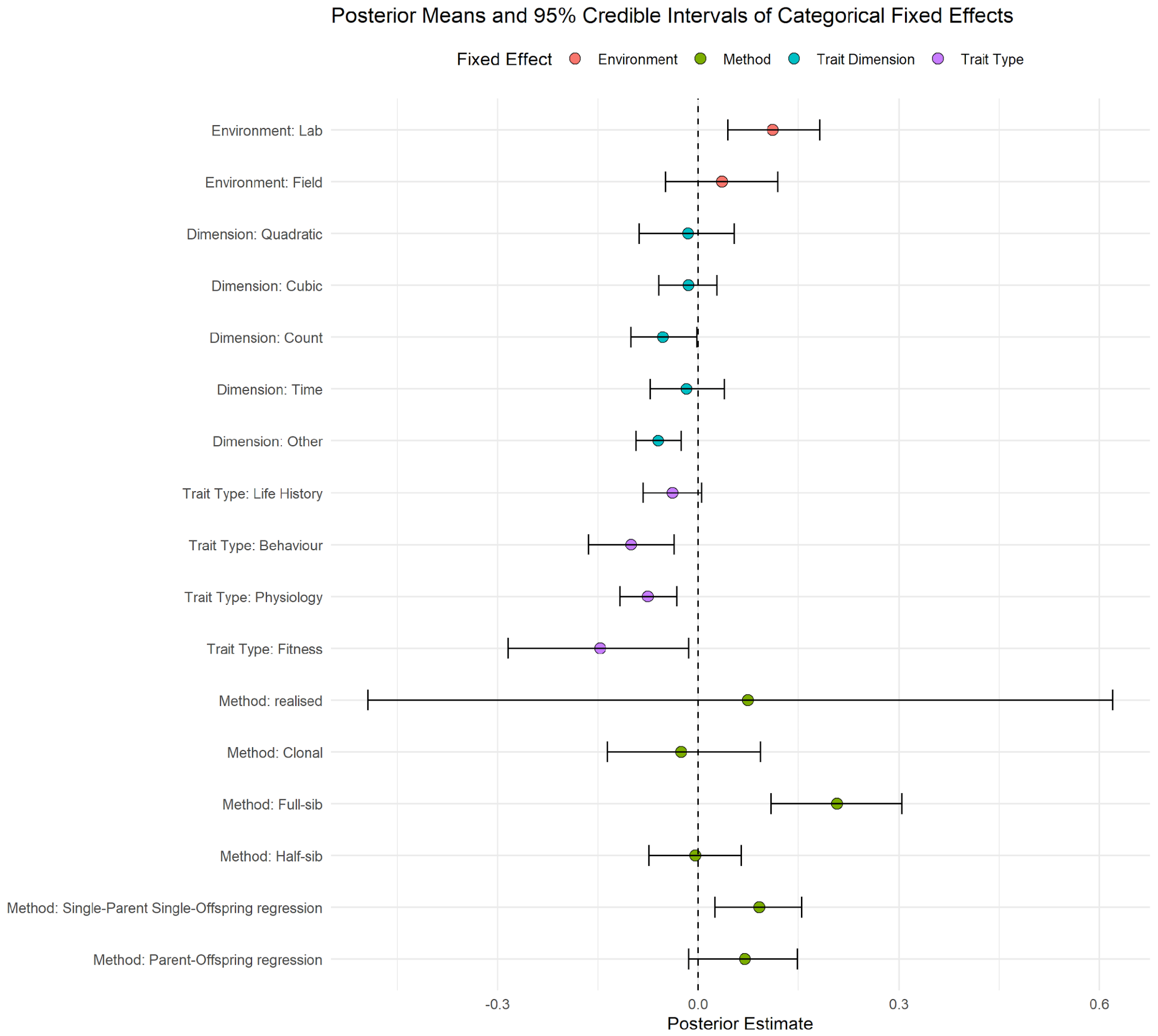

Fig. F: The posterior estimate for each fixed effect in the statistical model of h^2, along with its 95% credibility intervals; all estimates are deviations from the default model of a linear morphological trait estimated using an animal model from a population in the wild. The data underlying this figure can be found in the data repository accompanying this paper under the DOI: https://doi.org/10.5281/zenodo.20021814

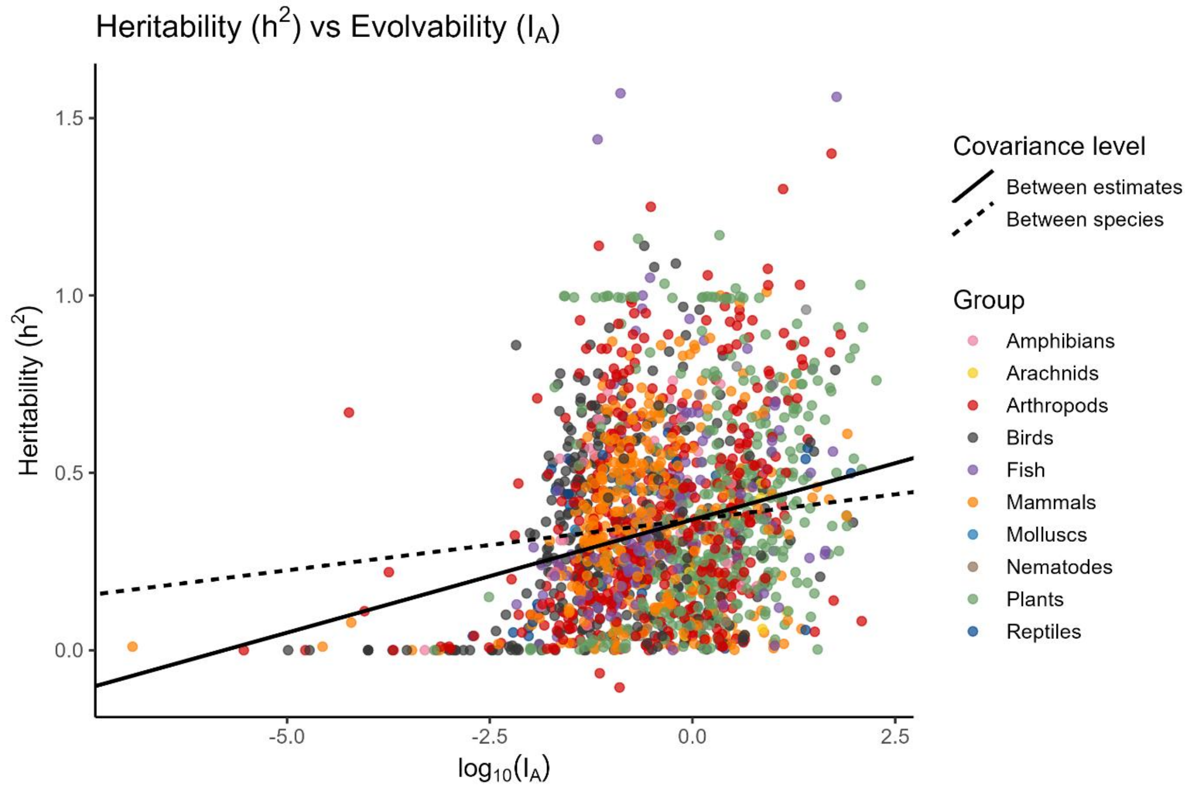

Fig. G: The relationship between evolvability (I_A) and heritability (h^2). Points show observed estimates by taxonomic group. Lines represent slopes derived from different variance-covariance components of the bivariate PGLMM. The between species slope (dashed – phylogenetic +non-phylogenetic) and the between estimate (solid – residual) slope. Lines are anchored at the posterior mean heritability from the bivariate model; intercepts are shown for visual comparison only. The data underlying this figure can be found in the data repository accompanying this paper under the DOI: https://doi.org/10.5281/zenodo.20021814

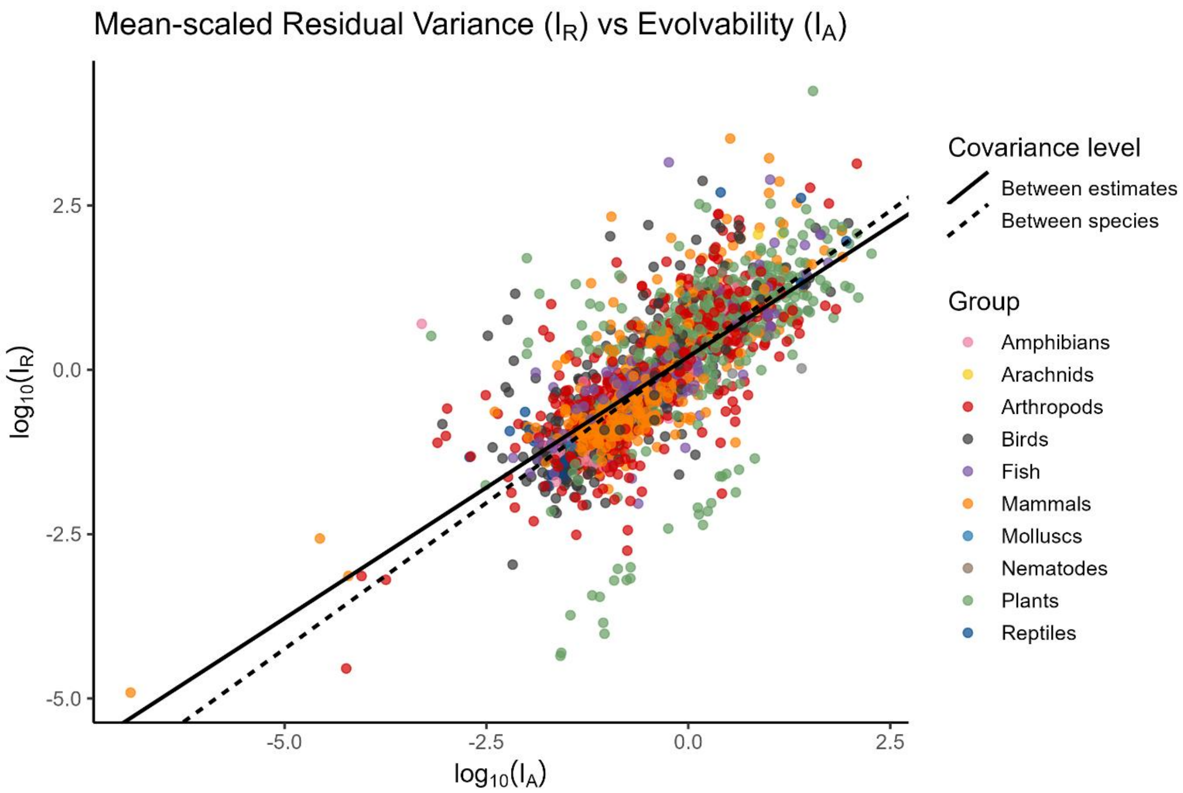

Fig. H: The relationship between evolvability (I_A) and the mean-scaled residual variance (I_R). Points show observed estimates by taxonomic group. Lines represent slopes derived from different variance-covariance components of the bivariate PGLMM. The between species slope (dashed – phylogenetic +non-phylogenetic) and the between estimate (solid – residual) slope. Lines are anchored at the posterior mean heritability from the bivariate model; intercepts are shown for visual comparison only. The data underlying this figure can be found in the data repository accompanying this paper under the DOI: https://doi.org/10.5281/zenodo.20021814

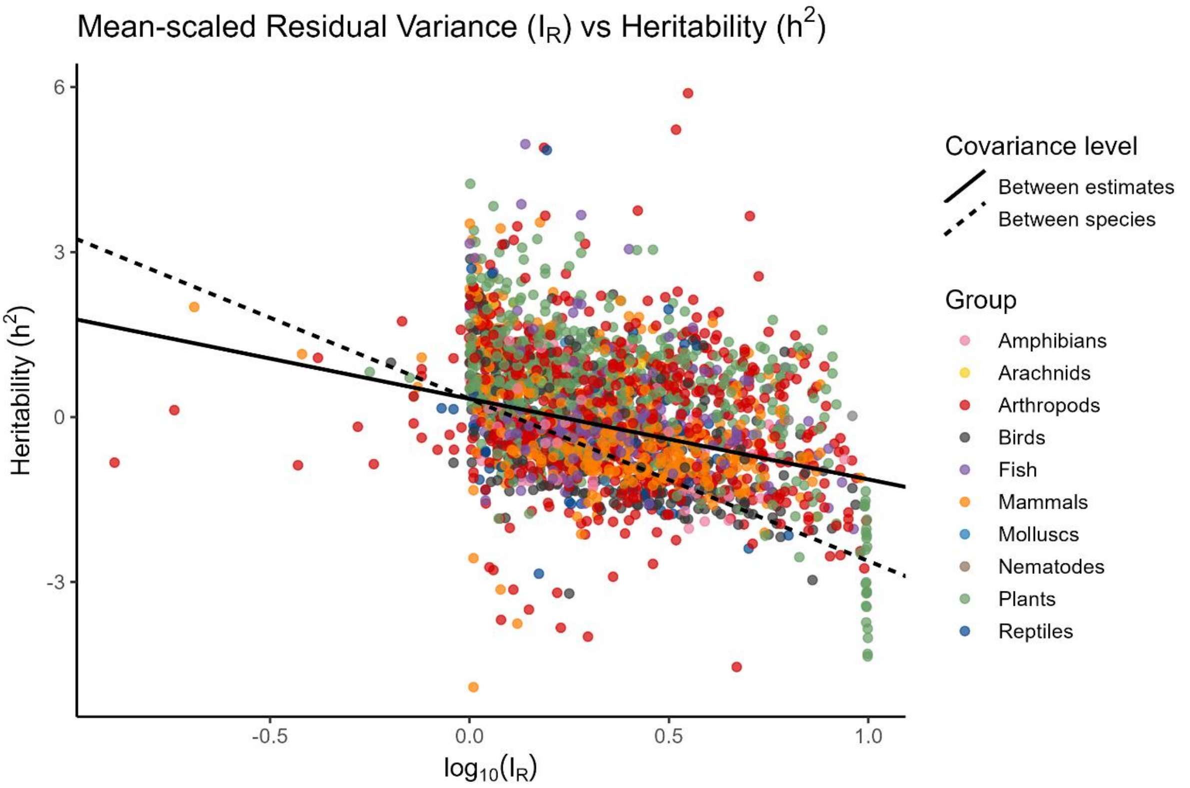

Fig. I: The relationship between heritability (h^2) and the mean-scaled residual variance (I_R). Points show observed estimates by taxonomic group. Lines represent slopes derived from different variance-covariance components of the bivariate PGLMM. The between species slope (dashed – phylogenetic +non-phylogenetic) and the between estimate (solid – residual) slope. Lines are anchored at the posterior mean heritability from the bivariate model; intercepts are shown for visual comparison only. The data underlying this figure can be found in the data repository accompanying this paper under the DOI: https://doi.org/10.5281/zenodo.20021814

Table E: Summary of the random and fixed effects for the interspecific variation model of the mean scaled residual variance (I_R). Random effects as the proportion of variance explained by them and the p-values of an omnibus test for the fixed effects.

Random Effect	mean	l-95%	u-95%	Percentage variance explained
**Phylogeny**	0.291	0.018	0.662	27% [5.9, 51]
**Non-Phylogenetic**	0.015	2e-08	0.054	1.6% [0, 5.5]
**Publication_ID**	0.249	0.176	0.329	26% [15, 37]
**Trait_2**	0.236	0.190	0.288	24% [16, 32]
**Residual**	0.213	0.193	0.233	22% [14, 28]

	mean	l-95%	u-95%	pMCMC	P(>x²)
**Intercept**	-0.144	-0.798	0.553	0.634	
**Method: clonal**	-0.100	-0.589	0.338	0.672	0.101
**Method: full-sib**	0.303	-0.166	0.766	0.200	
**Method: half-sib**	-0.070	-0.350	0.177	0.610	
**Method: mid-parent-offspring**	-0.470	-0.859	-0.113	0.017	
**Method: realized**	-0.848	-2.594	1.040	0.359	
**Method: single-parent-offspring**	-0.240	-0.550	0.039	0.104	
**Trait type: behaviour**	1.079	0.755	1.383	<4e-04	<4e-04
**Trait type: fitness**	1.045	0.445	1.636	<4e-04	
**Trait type: life history**	0.354	0.086	0.615	0.007	
**Trait type: physiology**	0.395	0.172	0.634	<4e-04	
**Dimension: quadratic**	0.281	-0.026	0.568	0.072	<4e-04
**Dimension: cubic**	0.620	0.415	0.832	<4e-04	
**Dimension: time**	0.350	0.032	0.636	0.033	
**Dimension: count**	0.585	0.353	0.828	<4e-04	
**Dimension: other**	0.352	0.104	0.601	0.008	
**n_fixed**	-0.005	-0.047	0.039	0.800	0.812
**n_random**	0.125	0.025	0.219	0.010	0.014
**Environment: lab**	-0.166	-0.491	0.180	0.331	0.624
**Environment: field/lab**	-0.053	-0.307	0.197	0.665	

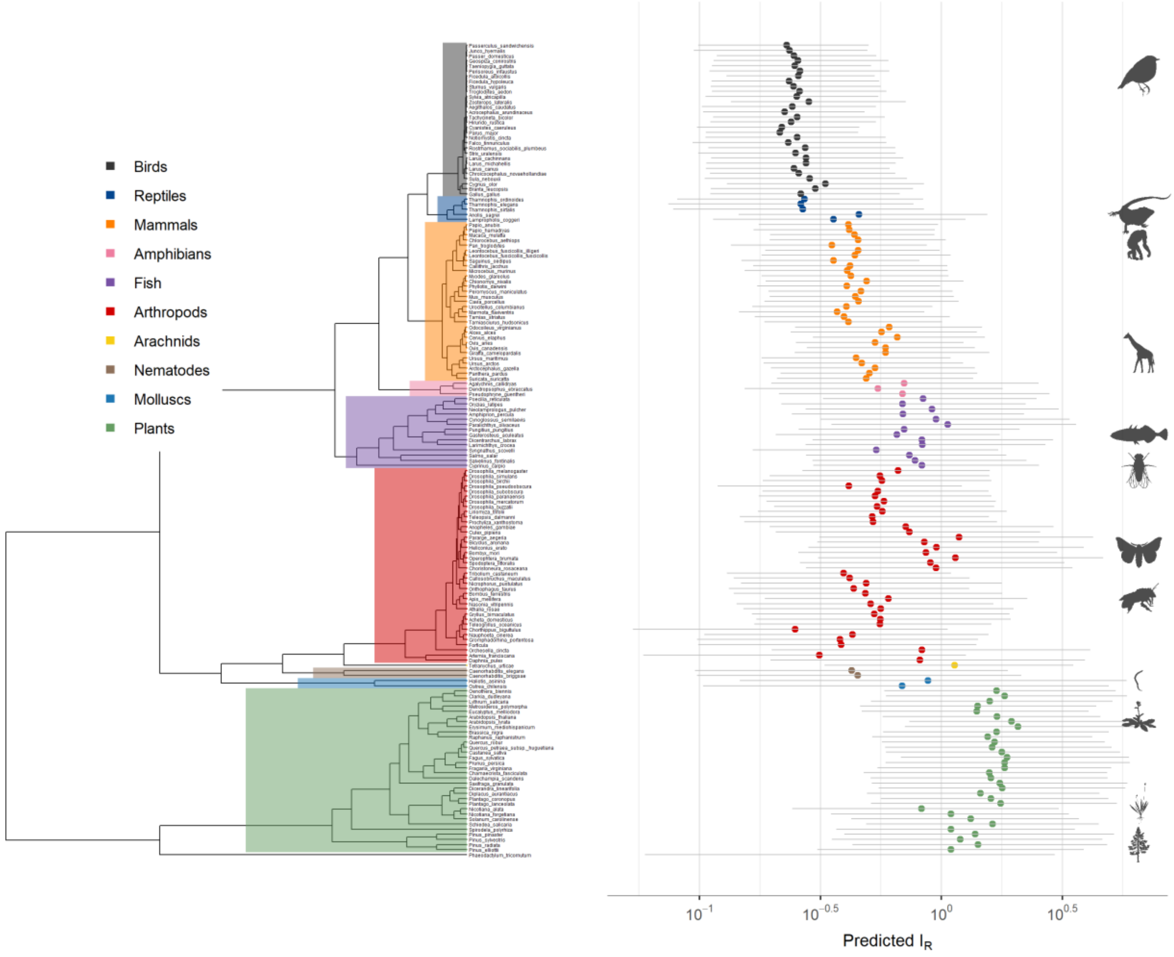

Fig. J: The predicted I_R (mean scaled residual variance) from our model for a linear, morphological trait estimated with an animal model from a wild population for each species, plotted across the phylogeny with their 95% credible intervals. Organism silhouettes were obtained from PhyloPic (https://www.phylopic.org) and are in the public domain (CC0 1.0 Universal Public Domain Dedication or Public Domain Mark 1.0). The data underlying this figure can be found in the data repository accompanying this paper under the DOI: https://doi.org/10.5281/zenodo.20021814
