# Supplementary material for: Levels of additive genetic variation vary substantially between species
Source: PLoS Biol. 2026 Jun 18;24(6):e3003819. doi: 10.1371/journal.pbio.3003819 (PMC13278422; doi:10.1371/journal.pbio.3003819)
Supplement: Attachment — Submitted filename: Response to Reviewers.docx [file pbio.3003819.s006.docx]

**Reviewer #1:**

This manuscript describes an impressive survey of the heritability and evolvability of quantitative traits in wild populations. The authors collect a comprehensive dataset, and then show that these fundamental quantitative genetic parameters vary between species, in a way that is explained by phylogeny; plants have especially high evolvability. They then go on to test whether various features that might be expected to affect additive genetic variation, such as mutation rate, genome size, and recombination rate influence h² and IA. They don't find evidence for any of these features being important, but discuss these findings in a thoughtful and balanced way.

I greatly enjoyed reading this manuscript. It's exceptionally clearly written and explained, and it tackles some big questions in evolutionary genetics. It seems almost certain to be very widely cited, and I think it would be a really good PLoS Biology paper. I have some suggestions (below), but they are really very minor.

Main Comments

The literature search is very well described and I can see why you took the decision to restrict the dataset to papers published in those four journals (JEB, Evolution, Heredity, Proc R Soc. B). However, given that one of the conclusions is that the sample sizes might be too small to detect the influence of, say, mutation rate, on h² and IA, that got me wondering what proportion of suitable studies had been captured by those four journals? It should be possible to crudely estimate this, even from the initial filtering of possible papers. It might be useful to discuss briefly what proportion of suitable studies you've sampled.

We thank the reviewer for their thoughtful comments. To address the question of how many potentially suitable studies our dataset captures, we conducted an additional analysis using the Web of Science (WoS) and a sequential filtering procedure designed to approximate the number of publications in the broader literature from which relevant estimates could plausibly be drawn. We focus on heritability estimates since the searches for this keyword are more refined. However, we note that almost all evolvability estimates are from papers that also quote a heritability estimate and hence our estimate for heritability should apply to evolvabilityd. Our approach, detailed in the supplementary text suggests that we have probably sampled only 20% of all estimates.

However, a more consequential limitation for our predictor analyses is not the absolute number of estimates but the number of species that we could compile heritability and evolvability for, which is much harder to quantify. Because this species-level coverage determines the overlap between our quantitative genetic measures (I_A_ and h^2^) and the predictor variables (e.g. mutation rate) we used targeted searches, designed to explicitly maximise that overlap. As a result, we expect that we captured the majority of published estimates available for these focal species at the time of sampling. We realize that these targeted searches were not originally mentioned in the manuscript and we have therefore added it into the manuscript now.

Minor comments and typos

Lines 69. 74. Is there a strong justification for choosing proteome size over exome size? Line 183 suggests you mean exome size anyway? I tend to think of the proteome as including all splice variants, whereas the exome refers to all coding parts of the genome. i.e. the former would count some sites multiple times, if they contribute to different splice variants, but the latter would count each site only one.

Our use of the term proteome size follows the definition employed by Lynch *et al*. (2023), which is where the data on this predictor originates. They quantify proteome size as “the total number of nucleotides employed in protein-coding genes (including alternatively spliced exons in species in which this occurs)”, which we believe is how the referee is defining exome size. We have now extended the relevant sentence to avoid any confusions on how we define proteome size.

Lines 125-126. Does removing the values that had zero or negative evolvability introduce a bias. i.e. by pruning the lowest values from the dataset.

Removing zero and negative evolvability estimates (n=146) is indeed a potential source of bias, as these values are disproportionately drawn from the lower tail of the distribution. We considered an alternative approach: fitting a censored model that retains these values by treating them as falling below a threshold. We implemented this in an earlier version of the analysis, and the results were qualitatively similar - the interspecific variation had credible intervals that did not cross zero, and the phylogenetic pattern in Figure 1 was upheld. However, the model fit of the censored approach was substantially worse than that of the final models, and because the zeroes and negative values (n=146) represented only 7% of all suitable data we proceeded with these models, removing non-positive estimates.

As the removal of undefined values removes the long-left tail of the I_A_ distribution, we are capping the lower end of the I_A_ estimates and underestimating the variation at the lower tail of the I_A_ distribution. While this might mean that we have potentially underestimated the variation in evolvability, it is unlikely to change any of our results fundamentally.

Page 229 - on the logarithm, not of the logarithm?

and

Page 233 - we fitted?

We thank the reviewer for spotting these and have changed this in the manuscript as suggested.

**Reviewer #2:**

This is a rather densely-packed ms that took a couple of reads. In large part, this is not because of poor writing/presentation, but rather that the meta-analysis attempts to examine so many features that there are a lot of moving parts to follow. The key features are both the size/scale of the dataset and the use of a phylogenetic mixed-model to allow for correlations between taxa (based on the inverse of their branch lengths). The ms attempts to model both narrow-sense heritability (h2) and evolvability (basically, differences in how the additive variance is scaled) and finds that there is little correlation between the two measures. Further, evolvability shows much greater variance than heritability. Most of my issues with the ms are minor, but one point of concern that needs to be addressed by the authors is that log(evolvability) was parameter modeled while heritability was modeled as unscaled. Further, heritability is constrained to lie within [0,1], resulting in compression and hence the potential for lower power. Hence, I think a few comments on this are in order in the final (and minor) revision.

We thank the reviewer for their insightful comments. The asymmetry in how the two response variables are modelled was a deliberate choice driven by the distributional properties of each metric, and we now explicitly note in the Methods that I_A_ and h^2^ results are not directly comparable. We also emphasise that we intentionally avoid treating them as if they were. In addition, we have added that the bounded [0,1] scale of h^2^ restricts the total variance available to be explained, which likely reduces statistical power relative to the I_A_ analysis.

Comments/minor points

Ln 47: Houle indeed has been the driving force behind evolvability, but Brian Charlesworth was the first to suggest this scaling (Charlesworth, B. 1984. The evolutionary genetics of life

histories. In B. Shorrocks (ed.), Evolutionary ecology, pp. 117-133. Blackwell Scientific, Oxford, UK.)

We have amended this in the manuscript to reflect that Brian Charlesworth was the first to suggest mean-scaling and that Houle then developed the concept.

ln 68: mutation rate* proteome size. The GWAS data seems to show that almost all GWAS hits are outside of coding regions (see Chapter 21 Walsh/Visscher/Lynch 2025)

We have added this to our discussion on reasons for why we don't observe any significant correlations with our predictors.

ln 80: nice estimate of Ne (diversity/mutation rate)!

We appreciate the reviewer’s positive feedback regarding the estimate of *N*e. This approach originates from work by Lewin and Eyre‑Walker (2026), who are pleased to see it recognised.

ln 109: Akesson seems to be missing from the ref list

Thank you for spotting this. Akesson reference has been added to the reference list

ln 107: I would use "mixed model" here (and elsewhere; ln 228) in place of "animal model" to avoid confusion for some

Thank you for this thoughtful suggestion. We appreciated the concern about potential confusion in terminology. In this context, however we intentionally use the term animal model because it refers specifically to the quantitative-genetic framework employed in the source paper. Many full-sib and half-sib studies also rely on mixed models, so replacing animal model with mixed model could reduce clarity about the specific methodological approach we used. For this reason, we retain the original terminology.

ln 148 (top right cell in Table 1). Walsh & Lynch (1998) should either be Lynch & Walsh (1998) or Walsh et al. (2025), see below.

Thank you very much for pointing this out. We had amended the reference to reflect the Lynch and Walsh (1998) reference.

ln 324-325: On negative estimates of h2, LS forces these to zero, while REML often avoids this. Might be interested to redo the heritability just using REML estimates. Another issue with REML is that which fixed factors are incorporated is important, as these can shrink the residual variance used to estimate h2 (sum of the variance components makes up the denominator, not the phenotypic variance). This can inflate estimates of h2 over those based on LS where these fixed-effect differences (e.g., sex differences) generating variation are folded into the phenotypic variance.

We agree that LS and REML methods can yield systematically different V_A_ estimates. We note that our estimation method is already included as a fixed effect in all models, which partially accounts for systematic differences between methods. However, we did not record whether individual estimates were derived using LS or REML and therefore cannot distinguish between the two methods in our dataset. Restricting the analysis to REML estimates only would therefore not be straightforward. Additionally, such a restriction would substantially reduce the number of estimates available for analysis. Any changes in results under the REML-only analysis would therefore be difficult to attribute to the statistical method per se rather than to the loss of statistical power resulting from the smaller dataset.

ln 411: additive variance scaling with Ne. A little tricky (e.g. WL pg 438), a point made by the senior author in earlier papers: The mutational variance is typically estimated with inbred lines, so that Ne is very small, and hence selection (s) against new mutations has to be large for them to be non-neutral. As Ne increases, Neu*, where u* is the effective neutral mutation rate, may not increase, as u* decreases with Ne (as 4Ne|s| increases, making fewer mutations neutral).

and

Ln 431—437: Same comment as for ln 411

We thank the reviewer for raising this point. We have now incorporated this point into the revised discussion. Specifically, we highlight that the expected relationship between V_A_ and N_e_ under neutrality or mutation-selection -drift balance is well established (Clayton and Robertson, 1955; Latter 1970; Bürger et al., 1989), and that the contribution of mutational variance to standing V_A_ depends on the fraction of mutations that are effectively neutral, which decreases as N_e_ increases (Walsh and Lynch, 2018). We also refer to recent work by Abson et al. (2026), who reframe these classical results in terms of nucleotide diversity and emphasize that strong V_A_~N_e_ scaling is only expected when traits behave neutrally or when Ne is sufficiently small that drift overwhelms selection. Together, these considerations strengthen the argument that a strong V_A_~N_e_ relationship is not generally expected, and we now emphasize this point when interpreting the lack of correlation in our analysis.

Ln 465-505: The work on crops typically finds only very small amounts of trait variation associated with cpDNA. I would argue that the plant feature a lot of evolutionary biologists overlook is meristematic tissues. Somatic mutations have no impact on offspring in animals, but a mutation occurring in a plant stem could impact its phenotype, such as flowers or leaf mass, (and hence be selected) with the gene tissue being downstream of that mutation, so there is some exposure to selection of new mutations before they are expressed in the offspring.

We agree that the organisation of meristematic tissues and the lack of an early segregated germline in plants is an important distinction from animals. However, the process highlighted would increase intra-organismal selection on mutations, which is expected to erode additive genetic variance rather than increase it. Since our manuscript focuses on explaining why plants show higher mean-scaled additive genetic variance, this mechanism does not align with the patterns we aim to explain.

We also appreciate the reviewer’s comments regarding cpDNA. As discussed in our subsequent correspondence with the reviewer, we have further amended this section in the manuscript and added references supporting substantial effects of organellar DNA on phenotypic variation in plants.

Ln 735 some confusion, largely due to me! Lynch & Walsh (1998) is correctly cited in the ms, but this is listed as Walsh & Lynch (1998) in the reference list. For L&W, the publisher is Sinauer, not Oxford. We just released Part I of the revision of the 1998 book, which is now Walsh, Visscher, and Lynch. 2025. Genetics and Analysis of Quantitative Traits: Foundations. Oxford. This part of the revision only covers the first part of the 1998 book. Hence the Ln 111 and 120 refs is correct (this material is not in Part I of the revision), but the ln 148 refs (above) could be either the 1998 or the 2025. Sorry for the confusion, there was just too much new stuff to fit the revision into a single volume.

Thank you very much for this clarification. We have amended this reference throughout and the reference list.

Bruce Walsh (in case you had not guessed)

12 Mar 2026
