# Supplementary material for: Levels of additive genetic variation vary substantially between species
Source: PLoS Biol. 2026 Jun 18;24(6):e3003819. doi: 10.1371/journal.pbio.3003819 (PMC13278422; doi:10.1371/journal.pbio.3003819)
Supplement: Peer review history

# Editor report: Decision Letter 0

**DOI:** 10.1371/journal.pbio.3003819.r001

**Authors:** Roland Roberts

20 Feb 2026

Dear Dr Zijmers,

Thank you for submitting your manuscript entitled "Levels of additive genetic variation vary substantially between species" for consideration as a Research Article by PLOS Biology.

Your Appeal of our previous decision to decline your manuscript has now been evaluated by the PLOS Biology editorial staff, as well as by an academic editor with relevant expertise, and I'm writing to let you know that we would like to send your submission out for external peer review.

However, before we can send your manuscript to reviewers, we need you to complete your submission by providing the metadata that is required for full assessment. To this end, please login to Editorial Manager where you will find the paper in the 'Submissions Needing Revisions' folder on your homepage. Please click 'Revise Submission' from the Action Links and complete all additional questions in the submission questionnaire.

Once your full submission is complete, your paper will undergo a series of checks in preparation for peer review. After your manuscript has passed the checks it will be sent out for review. To provide the metadata for your submission, please Login to Editorial Manager (https://www.editorialmanager.com/pbiology) within two working days, i.e. by Feb 24 2026 11:59PM.

If your manuscript has been previously peer-reviewed at another journal, PLOS Biology is willing to work with those reviews in order to avoid re-starting the process. Submission of the previous reviews is entirely optional and our ability to use them effectively will depend on the willingness of the previous journal to confirm the content of the reports and share the reviewer identities. Please note that we reserve the right to invite additional reviewers if we consider that additional/independent reviewers are needed, although we aim to avoid this as far as possible. In our experience, working with previous reviews does save time.

If you would like us to consider previous reviewer reports, please edit your cover letter to let us know and include the name of the journal where the work was previously considered and the manuscript ID it was given. In addition, please upload a response to the reviews as a 'Prior Peer Review' file type, which should include the reports in full and a point-by-point reply detailing how you have or plan to address the reviewers' concerns.

During the process of completing your manuscript submission, you will be invited to opt-in to posting your pre-review manuscript as a bioRxiv preprint. Visit http://journals.plos.org/plosbiology/s/preprints for full details. If you consent to posting your current manuscript as a preprint, please upload a single Preprint PDF.

Feel free to email us at plosbiology@plos.org if you have any queries relating to your submission.

Kind regards,

Roli Roberts

Roland Roberts, PhD

Senior Editor

PLOS Biology

rroberts@plos.org

# Peer review: Decision Letter 1

**DOI:** 10.1371/journal.pbio.3003819.r002

**Authors:** Roland Roberts

21 Mar 2026

Dear Dr Zijmers,

Thank you for your patience while your manuscript "Levels of additive genetic variation vary substantially between species" was peer-reviewed at PLOS Biology. It has now been evaluated by the PLOS Biology editors, an Academic Editor with relevant expertise, and by two independent reviewers.

Based on the reviews, we are likely to accept this manuscript for publication, provided you satisfactorily address the remaining points raised by the reviewers and the following data and other policy-related requests.

IMPORTANT - please attend to the following:

a) Please address the requests from the reviewers. You'll see that Reviewer #1 is very positive, but wants you to estimate the proportion of studies which your search strategy captures, and has a short list of minor points. Reviewer #2 doesn’t assess the overall advance, but sounds positive, and has a somewhat longer list of relatively minor points, one of which involves further (minor) analysis.

b) Please address my Data Policy requests below; specifically, we need you to supply the numerical values underlying Figs 1, 2, 3, S1, S2, S3, S4, S5, S6, S7, S8, S9, S10, either as a supplementary data file or as a permanent DOI’d deposition. I note that you have stated intention to deposit these in Zenodo. Please can you now do so (I will need to check data policy compliance before accepting), and confirm whether the data and code in this deposition are sufficient to recreate the Figures?

c) Please cite the location of the data clearly in all relevant main and supplementary Figure legends, e.g. “The data and code needed to generate this Figure can be found in https://zenodo.org/records/XXXXXXXX" and update your Data Availability statement accordingly.

d) Please include the URL of your funder in the Financial Disclosure statement.

As you address these items, please take this last chance to review your reference list to ensure that it is complete and correct. If you have cited papers that have been retracted, please include the rationale for doing so in the manuscript text, or remove these references and replace them with relevant current references. Any changes to the reference list should be mentioned in the cover letter that accompanies your revised manuscript.

In addition to these revisions, you may need to complete some formatting changes, which you will receive in a follow up email. A member of our team will be in touch with a set of requests shortly. If you do not receive a separate email within a few days, please assume that checks have been completed, and no additional changes are required.

We expect to receive your revised manuscript within two weeks.

To submit your revision, please go to https://www.editorialmanager.com/pbiology/ and log in as an Author. Click the link labelled 'Submissions Needing Revision' to find your submission record. Your revised submission must include the following:

- a cover letter that should detail your responses to any editorial requests, if applicable, and whether changes have been made to the reference list

- a Response to Reviewers file that provides a detailed response to the reviewers' comments (if applicable, if not applicable please do not delete your existing 'Response to Reviewers' file.)

- a track-changes file indicating any changes that you have made to the manuscript.

NOTE: If Supporting Information files are included with your article, note that these are not copyedited and will be published as they are submitted. Please ensure that these files are legible and of high quality (at least 300 dpi) in an easily accessible file format. For this reason, please be aware that any references listed in an SI file will not be indexed. For more information, see our Supporting Information guidelines:

https://journals.plos.org/plosbiology/s/supporting-information

*Published Peer Review History*

Please note that you may have the opportunity to make the peer review history publicly available. The record will include editor decision letters (with reviews) and your responses to reviewer comments. If eligible, we will contact you to opt in or out. Please see here for more details:

https://plos.org/published-peer-review-history/

*Press*

Should you, your institution's press office or the journal office choose to press release your paper, please ensure you have opted out of Early Article Posting on the submission form. We ask that you notify us as soon as possible if you or your institution is planning to press release the article.

*Protocols deposition*

To enhance the reproducibility of your results, we recommend that if applicable you deposit your laboratory protocols in protocols.io, where a protocol can be assigned its own identifier (DOI) such that it can be cited independently in the future. Additionally, PLOS ONE offers an option for publishing peer-reviewed Lab Protocol articles, which describe protocols hosted on protocols.io. Read more information on sharing protocols at https://plos.org/protocols?utm_medium=editorial-email&utm_source=authorletters&utm_campaign=protocols

Please do not hesitate to contact me should you have any questions.

Sincerely,

Roli Roberts

Roland Roberts, PhD

Senior Editor

rroberts@plos.org

PLOS Biology

------------------------------------------------------------------------

DATA POLICY:

You may be aware of the PLOS Data Policy, which requires that all data be made available without restriction: http://journals.plos.org/plosbiology/s/data-availability. For more information, please also see this editorial: http://dx.doi.org/10.1371/journal.pbio.1001797

Note that we do not require all raw data. Rather, we ask that all individual quantitative observations that underlie the data summarized in the figures and results of your paper be made available in one of the following forms:

1) Supplementary files (e.g., excel). Please ensure that all data files are uploaded as 'Supporting Information' and are invariably referred to (in the manuscript, figure legends, and the Description field when uploading your files) using the following format verbatim: S1 Data, S2 Data, etc. Multiple panels of a single or even several figures can be included as multiple sheets in one excel file that is saved using exactly the following convention: S1_Data.xlsx (using an underscore).

2) Deposition in a publicly available repository. Please also provide the accession code or a reviewer link so that we may view your data before publication.

Regardless of the method selected, please ensure that you provide the individual numerical values that underlie the summary data displayed in the following figure panels as they are essential for readers to assess your analysis and to reproduce it: Figs 1, 2, 3, S1, S2, S3, S4, S5, S6, S7, S8, S9, S10. NOTE: the numerical data provided should include all replicates AND the way in which the plotted mean and errors were derived (it should not present only the mean/average values).

IMPORTANT: Please also ensure that figure legends in your manuscript include information on where the underlying data can be found, and ensure your supplemental data file/s has a legend.

Please ensure that your Data Statement in the submission system accurately describes where your data can be found.

------------------------------------------------------------------------

CODE POLICY

Per journal policy, if you have generated any custom code during the course of this investigation, please make it available without restrictions. Please ensure that the code is sufficiently well documented and reusable, and that your Data Statement in the Editorial Manager submission system accurately describes where your code can be found. More information on our Code Policy, what and how to share can be found here: https://journals.plos.org/plosbiology/s/code-availability

Please note that we cannot accept sole deposition of code in GitHub, as this could be changed after publication. However, you can archive this version of your publicly available GitHub code to Zenodo. Once you do this, it will generate a DOI number, which you will need to provide in the Data Accessibility Statement (you are welcome to also provide the GitHub access information). See the process for doing this here: https://docs.github.com/en/repositories/archiving-a-github-repository/referencing-and-citing-content

------------------------------------------------------------------------

DATA NOT SHOWN?

- Please note that per journal policy, we do not allow the mention of "data not shown", "personal communication", "manuscript in preparation" or other references to data that is not publicly available or contained within this manuscript. Please either remove mention of these data or provide figures presenting the results and the data underlying the figure(s).

------------------------------------------------------------------------

REVIEWERS' COMMENTS:

Reviewer #1:

This manuscript describes an impressive survey of the heritability and evolvability of quantitative traits in wild populations. The authors collect a comprehensive dataset, and then show that these fundamental quantitative genetic parameters vary between species, in a way that is explained by phylogeny; plants have especially high evolvability. They then go on to test whether various features that might be expected to affect additive genetic variation, such as mutation rate, genome size, and recombination rate influence h² and IA. They don't find evidence for any of these features being important, but discuss these findings in a thoughtful and balanced way.

I greatly enjoyed reading this manuscript. It's exceptionally clearly written and explained, and it tackles some big questions in evolutionary genetics. It seems almost certain to be very widely cited, and I think it would be a really good PLoS Biology paper. I have some suggestions (below), but they are really very minor.

Main Comments

The literature search is very well described and I can see why you took the decision to restrict the dataset to papers published in those four journals (JEB, Evolution, Heredity, Proc R Soc. B). However, given that one of the conclusions is that the sample sizes might be too small to detect the influence of, say, mutation rate, on h² and IA, that got me wondering what proportion of suitable studies had been captured by those four journals? It should be possible to crudely estimate this, even from the initial filtering of possible papers. It might be useful to discuss briefly what proportion of suitable studies you've sampled.

Minor comments and typos

Lines 69. 74. Is there a strong justification for choosing proteome size over exome size? Line 183 suggests you mean exome size anyway? I tend to think of the proteome as including all splice variants, whereas the exome refers to all coding parts of the genome. i.e. the former would count some sites multiple times, if they contribute to different splice variants, but the latter would count each site only one.

Lines125-126. Does removing the values that had zero or negative evolvability introduce a bias. i.e. by pruning the lowest values from the dataset.

Page 229 - on the logarithm, not of the logarithm?

Page 233 - we fitted?

Reviewer #2:

This is a rather densely-packed ms that took a couple of reads. In large part, this is not because of poor writing/presentation, but rather that the meta-analysis attempts to examine so many features that there are a lot of moving parts to follow. The key features are both the size/scale of the dataset and the use of a phylogenetic mixed-model to allow for correlations between taxa (based on the inverse of their branch lengths). The ms attempts to model both narrow-sense heritability (h2) and evolvability (basically, differences in how the additive variance is scaled), and finds that there is little correlation between the two measures. Further, evolvability shows much greater variance than heritability. Most of my issues with the ms are minor, but one point of concern that needs to be addressed by the authors is that log(evolvability) was parameter modeled while heritability was modeled as unscaled. Further, heritability is constrained to lie within [0,1], resulting in compression and hence the potential for lower power. Hence, I think a few comments on this are in order in the final (and minor) revision.

Comments/minor points

Ln 47: Houle indeed has been the driving force behind evolvability, but Brian Charlesworth was the first to suggest this scaling (Charlesworth, B. 1984. The evolutionary genetics of life

histories. In B. Shorrocks (ed.), Evolutionary ecology, pp. 117-133. Blackwell Scientific, Oxford, UK.)

ln 68: mutation rate* proteome size. The GWAS data seems to show that almost all GWAS hits are outside of coding regions (see Chapter 21 Walsh/Visscher/Lynch 2025)

ln 80: nice estimate of Ne (diversity/mutation rate)!

ln 109: Akesson seems to be missing from the ref list

ln 107: I would use "mixed model" here (and elsewhere; ln 228) in place of "animal model" to avoid confusion for some

ln 148 (top right cell in Table 1). Walsh & Lynch (1998) should either be Lynch & Walsh (1998) or Walsh et al. (2025), see below.

ln 324-325: On negative estimates of h2, LS forces these to zero, while REML often avoids this. Might be interested to redo the heritability just using REML estimates. Another issue with REML is that which fixed factors are incorporated is important, as these can shrink the residual variance used to estimate h2 (sum of the variance components makes up the denominator, not the phenotypic variance). This can inflate estimates of h2 over those based on LS where these fixed-effect differences (e.g., sex differences) generating variation are folded into the phenotypic variance.

ln 411: additive variance scaling with Ne. A little tricky (e.g. WL pg 438), a point made by the senior author in earlier papers: The mutational variance is typically estimated with inbred lines, so that Ne is very small, and hence selection (s) against new mutations has to be large for them to be non-neutral. As Ne increases, Neu*, where u* is the effective neutral mutation rate, may not increase, as u* decreases with Ne (as 4Ne|s| increases, making fewer mutations neutral).

Ln 431—437: Same comment as for ln 411

Ln 465-505: The work on crops typically finds only very small amounts of trait variation associated with cpDNA. I would argue that the plant feature a lot of evolutionary biologists overlook is meristematic tissues. Somatic mutations have no impact on offspring in animals, but a mutation occurring in a plant stem could impact its phenotype, such as flowers or leaf mass, (and hence be selected) with the gene tissue being downstream of that mutation, so there is some exposure to selection of new mutations before they are expressed in the offspring.

Ln 735 some confusion, largely due to me! Lynch & Walsh (1998) is correctly sited in the ms, but this is listed as Walsh & Lynch (1998) in the reference list. For L&W, the publisher is Sinauer, not Oxford. We just released Part I of the revision of the 1998 book, which is now Walsh, Visscher, and Lynch. 2025. Genetics and Analysis of Quantitative Traits: Foundations. Oxford. This part of the revision only covers the first part of the 1998 book. Hence the Ln 111 and 120 refs is correct (this maternal is not in Part I of the revision), but the ln 148 refs (above) could be either the 1998 or the 2025. Sorry for the confusion, there was just too much new stuff to fit the revision into a single volume.

Bruce Walsh (in case you had not guessed)

12 Mar 2026

# Author comment: Author response to Decision Letter 2

**DOI:** 10.1371/journal.pbio.3003819.r003

7 May 2026

Attachment Submitted filename: Response to Reviewers.docx

# Editor report: Decision Letter 2

**DOI:** 10.1371/journal.pbio.3003819.r004

**Authors:** Roland Roberts

8 May 2026

Dear Lillith,

Thank you for the submission of your revised Research Article "Levels of additive genetic variation vary substantially between species" for publication in PLOS Biology. On behalf of my colleagues and the Academic Editor, Nick Barton, I'm pleased to say that we can in principle accept your manuscript for publication, provided you address any remaining formatting and reporting issues. These will be detailed in an email you should receive within 2-3 business days from our colleagues in the journal operations team; no action is required from you until then. Please note that we will not be able to formally accept your manuscript and schedule it for publication until you have completed any requested changes.

Please take a minute to log into Editorial Manager at http://www.editorialmanager.com/pbiology/, click the "Update My Information" link at the top of the page, and update your user information to ensure an efficient production process.

PRESS: We frequently collaborate with press offices. If your institution or institutions have a press office, please notify them about your upcoming paper at this point, to enable them to help maximise its impact. If the press office is planning to promote your findings, we would be grateful if they could coordinate with biologypress@plos.org. If you have previously opted in to the early version process, we ask that you notify us immediately of any press plans so that we may opt out on your behalf.

We also ask that you take this opportunity to read our Embargo Policy regarding the discussion, promotion and media coverage of work that is yet to be published by PLOS. As your manuscript is not yet published, it is bound by the conditions of our Embargo Policy. Please be aware that this policy is in place both to ensure that any press coverage of your article is fully substantiated and to provide a direct link between such coverage and the published work. For full details of our Embargo Policy, please visit http://www.plos.org/about/media-inquiries/embargo-policy/.

Thank you again for choosing PLOS Biology for publication and supporting Open Access publishing. We look forward to publishing your study.

Sincerely,

Roli

Roland G Roberts, PhD, PhD

Senior Editor

PLOS Biology

rroberts@plos.org
